# The broken care chain—report from a country with a low number of hospital beds

**DOI:** 10.3389/frhs.2025.1632220

**Published:** 2025-09-25

**Authors:** Marie Jönsson, Peter Appelros, Marie Holmefur, Carin Fredriksson

**Affiliations:** Department of Health Sciences, Örebro University, Örebro, Sweden

**Keywords:** healthcare, healthcare professionals, older adults, phenomenography, readmission, social care

## Abstract

**Background:**

Approximately 30% of older adults admitted to hospital in Sweden are readmitted within three months. Short hospital stays and readmission can lead to further functional decline, as recovery appears to be poor at home after discharge.

**Aim:**

To explore healthcare professionals’ experiences of transitional care in older adults in order to prevent readmission.

**Methods:**

Four focus group interviews were conducted with healthcare professionals (*n* = 29). Data were analyzed using a phenomenographic approach.

**Results:**

Healthcare professionals’ perceptions were compiled into seven descriptive categories. Three of the categories, i.e., *resources*, *interprofessional coordination*, and *advanced care needs can be difficult to meet*, described healthcare professionals’ perceptions of the current stage of older adults—the first-order perspective, i.e., *what something is*. The remaining categories described the meanings of the healthcare professionals’ perceptions.

**Conclusion:**

Several interacting structural issues cause readmissions. These include premature discharge from hospital, poor hand-over between healthcare professionals, and a lack of qualified staff in the home-setting. To prevent readmission, medical competence and interprofessional teamwork must be improved in the home setting.

## Introduction

Efficient coordination between healthcare and social care is particularly important for older adults with complex health conditions such as multiple diagnoses, functional limitations, cognitive deficits, and social needs (1–3). Approximately 30% of individuals belonging to this group are readmitted to hospital within three months of a first inpatient stay caused by acute problems (4, 5). Readmission is associated with increased healthcare costs, length of hospital stay, and chronic disease, and with decreased well-being for the patients after discharge (5–10). To meet care needs and prevent readmission in this group of older adults, it is therefore of interest to study what factors are important for the quality of transitional care.

Many older adults prefer to age at home, but for this to be possible, they must be offered support and care at home. Reporting should therefore be highly effective when an older adult is discharged from hospital. In this context, we speak of transitional care (11, 12), defined as the care measures taken in the transitional phase between care in hospital and care at home. Several factors important for the quality of transitional care have been identified, such as healthcare professionals’ competence, the complexity of the patient's clinical picture, and the number of patients (13). Furthermore, patient participation, involvement of relatives, and multidisciplinary work can have a positive impact on establishing good and safe care at home (14–16). A central aspect of transitional care is the discharge planning process (DPP), which requires healthcare professionals from different organizations to cooperate and interact (2, 16). A discharge plan tailored to meeting individual needs seems to decrease the risk of early readmission (8, 16). Certain care interventions after discharge have been identified to prevent early readmission, such as home visits, medication reconciliation, patient education, dietary support, and follow-up appointments (17–20). Also, occupational therapy interventions focusing on activities of daily living (ADL) performance have been shown to prevent readmission in certain patient groups, probably by focusing on ability and social needs (21).

Because Sweden has the lowest per capita hospital bed rate in Europe (22), the implementation of the DPP is extremely important for the quality of homecare to be satisfactory. The time for DPP completion has decreased from six to three days in recent years (23). If the primary care and municipal care organizations are unable to receive a patient after three days, payment liability arises. As hospitals have a shortage of beds, and as municipalities want to avoid payment liability, in-hospital stay times and the DPP become very short (5, 23).

Despite efforts to improve transitional care, reports have concluded that there are still unmet needs related to caring issues, information, and social activities (24–26). The experiences of healthcare professionals are probably an underutilized resource in this context. Through daily observations, healthcare staff can observe shortcomings in the care chain. Such observations may identify areas for improvement, which can be used in ongoing quality work. We decided to conduct a qualitative study exploring healthcare professionals’ experiences of transitional care in older adults, with the aim of finding opportunities for improvement in the care organization to prevent readmissions.

## Methods

This study applies a descriptive qualitative design using a phenomenographic method inspired by Marton (27) and is based on data from four focus group interviews (FGIs) (28) with healthcare professionals. The phenomenographic method was chosen to capture variations in the participants’ perceptions related to the transitional care of older adults, particularly regarding how readmission can be prevented. The method has a pedagogical approach, and distinguishes between two perspectives when describing knowledge: the first-order perspective, i.e., what something is, and the second-order perspective, i.e., how something is perceived (27). The focus when using a phenomenographic method is to identify the second-order perspective and describe the various ways in which the participants experience, understand, or perceive a certain phenomenon in the world around them (27). The three expressions—*express*, *describe*, and *perceive*—are used synonymously in this study.

### Participants and procedure

Focus groups require that the participants should have something in common (homogeneity), but there should also be differences in some aspects (heterogeneity) in order to stimulate the group conversation (28). The following inclusion criteria were set: participants should have experience in assessing and treating older adults readmitted to hospital and represent a variety of healthcare professions in different settings, namely, acute hospital care, primary healthcare, and municipal social care. In this study, older adults are defined as persons aged 75 years or older (18, 29).

After initial contacts with the department heads, two managers in the hospital department of medicine, six managers of social services in two municipalities, and one manager in primary healthcare were sent information about the study via email. If there was no response, reminders were sent. As a third measure, phone calls were made. Managers who showed interest in the study were verbally informed and asked to identify healthcare professionals who met the inclusion criteria. Presumptive participants were sent an information letter and were also verbally informed by the first author. The participants originated from two acute medical hospital departments (around 45 beds), one large municipality (around 144,000 inhabitants), one smaller municipality (around 32,000 inhabitants), and one primary healthcare center (around 22,000 patients). The units were situated near the demographic center of Sweden (Table 1).

**Table 1:** Summarized description of the participants (focus groups, *n* = 4).

Focus group	Total, *n*	Sex, F/M, *n*	Healthcare professionals	Setting	Years of work experience, range
FG 1	9	8/1	Assistant nurses (*n* = 2) Healthcare coordinator (nurse) (*n* = 1) Discharge coordinators (nurse and assistant nurse) (*n* = 2) Nurses (*n* = 2) Occupational therapist (*n* = 1) Physiotherapist (*n* = 1)	Hospital	2–45
FG 2	6	4/2	Acute care physicians (*n* = 4) Physicians (*n* = 2)	Hospital and primary healthcare	6–40
FG 3	6	6/0	Assistant nurse (homecare services) (*n* = 1) Assistant nurse (home rehab) (*n* = 1) Discharge coordinator (nurse and occupational therapist) (*n* = 2) Occupational therapist (*n* = 1) Physiotherapist (*n* = 1)	Municipality	10–40
FG 4	8	8/0	Assistant nurses (homecare services) (*n* = 2) Discharge coordinator (nurse) (*n* = 1) Nurse (preventive care) (*n* = 1) Occupational therapists (*n* = 2) Physiotherapists (*n* = 2)	Municipality	10–29

### Data collection

The FGI method was chosen as group interactions may help participants to speak freely and express experiences, attitudes, and beliefs related to the studied phenomenon. Due to collective discussion and interaction among the participants, FGIs can provide new views and thoughts related to the studied phenomenon (28). The FGIs were conducted and moderated by the first author, with the last author present as an observer. The first author is an occupational therapist with experience in acute hospital care and at the time was a PhD student. Her previous research experience concerns older adults repeatedly readmitted to hospital.

An interview guide was formulated according to the principles of phenomenographic interviews (30) and the aim of the study. The following are examples of key questions:

• In your view, what do you perceive that older adults readmitted to hospital have usually been offered?
  ○ What difficulties do you perceive may exist when assessing older adults with repeated readmissions?
  ○ What difficulties do you perceive may exist when performing interventions for older adults with repeated readmissions?
• What do you perceive as effective (valuable) assessments and interventions in order to prevent readmissions?
  ○ How does your healthcare profession contribute to assessments and interventions in order to prevent readmissions?
• How do you perceive that assessments and interventions in healthcare and social care need to be developed to prevent the readmission of older adults?

The FGIs were performed during the participants’ working hours. All FGIs started with the moderator (first author) welcoming the participants and providing general information on how the group interview and discussion would be conducted. The participants then shared their experiences of working with older adults within their profession.

The observer made notes about the discussion, using them to support the data analyses. The first FGI was performed in a conference room, but due to pandemic-related restrictions, the following FGIs were held digitally. A total of 29 healthcare professionals, 26 women and three men, participated in four FGIs (Table 1). The FGI participants had two to 40 years of work experience. Three FGIs comprised assistant nurses, nurses, occupational therapists, and physiotherapists from acute hospital care in two different municipalities, whereas the other FGI comprised physicians from acute hospital care and primary healthcare (Table 1). Each FGI lasted 60–98 min (average 78 min) and was digitally recorded.

### Data analysis

The analysis of the FGI data was inspired by Dahlgren and Fallsberg's seven-step process (31). The first step (familiarization) started with making a word-for-word transcript of each FGI, which was coded and then transferred to NVivo 12 software (32). The transcripts were read several times by the first and last authors to obtain a sense of the whole. In the second step (condensation), statements relevant to the study were identified, read several times, and then condensed into meaning units to clarify the participants’ various perceptions of the phenomenon. In the third step (comparison), the condensed meaning units were compared in order to identify variations in the perceptions of the studied phenomena. Thereafter, in step four (grouping), the units were sorted and grouped into preliminary categories. The preliminary categories were directly related to the studied phenomena and reflected the participants’ perceptions. In step five (articulating), the first and the last authors compared statements in each category with the transcribed interviews to confirm that the data were well founded. This step was repeated several times. In step six (labeling), each category was labeled with a description that captured its essential meaning. In step seven (contrasting), the contents of the categories were compared for similarities and differences and discussed by all authors.

### Trustworthiness

In this study, credibility was confirmed through the first and last authors’ continuous engagement with the data. To assess the dependability of the data, the FGIs were listened to and the transcripts carefully read several times by the first author. To increase conformability, the processes of recruiting the participants, analyzing the data, and presenting the results were continuously discussed by the research team, constituting a form of triangulation (33).

### Ethical approval

The study was conducted in line with ethical principles for medical research on human subjects in accordance with the Declaration of Helsinki. The healthcare professionals received information about the study from their managers in the various care settings. Those who showed interest in participating were then given verbal and written information about the study by the first author, and their written consent was obtained. The study protocol was reviewed by the Swedish Ethical Review Authority, which found that the project did not fall under the purview of the Swedish Ethical Review Act but had no objections to it (*Blinded information*).

## Results

The analysis resulted in the identification of seven descriptive categories depicting different conceptions of what healthcare professionals said was important in hindering or delaying the readmission of older adults to hospital. Three of the categories, i.e., *resources*, *interprofessional coordination*, and *advanced care needs can be difficult to meet*, were described by the healthcare professionals as important in delaying readmission, i.e., the first-order ‘‘what’’ aspects. The remaining four categories described the various ways the participants talked about hindering/delaying readmission, i.e., the second-order ‘‘how’’ aspects.

**Figure 1:**
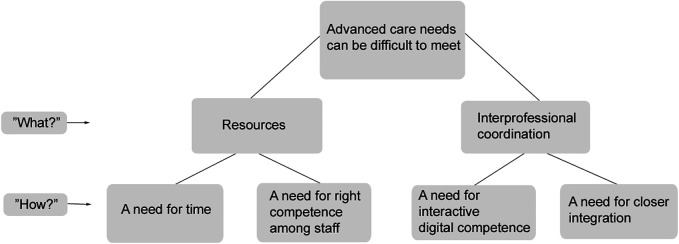
The outcome space presenting the categories and the healthcare professionals perceptions.

The logical relationship (i.e., the outcome space) among the categories is shown in Figure 1. The meanings of *resources* are described in the categories: *a need for time* and *a need for right competence among staff.* The meanings of *interpersonal coordination* are described in the categories: *a need for interactive digital systems* and *a need for closer integration*.

### Resources

In this category, the perception was that there were issues with the structure of healthcare and social care. The older adult may be suffering from diseases that are difficult to treat at home, and his/her state of health may be fragile upon returning home. The main problem, as perceived in the FGIs, was insufficient numbers of hospital beds, lack of time, and lack of personnel, all of which contributed to short hospital stays.

### A need for time

The short hospital stays due to the shortage of hospital beds and the DPP regulations were described as problematic. This resulted in premature discharge, which was perceived to increase the risk of readmission:

It takes an understanding physician to keep them in hospital those extra days to investigate the issue. This is also part of this [i.e., the healthcare system]. We can only keep them so many days in the hospital, so they come back again … They should not be sent home at once … And we actually have to stand up for our older adults. We can’t just send them home. (Discharge coordinator/nurse, Department of medicine, FG 1)

Healthcare professionals working in hospitals emphasized that different professions sometimes arrived at contradictory results concerning available hospital beds and discharge assessments. For example, the physician's assessment could show that an older adult was medically fit to leave hospital, whereas other professionals might assess the opposite:

I have to be strong enough to protest and say it is indefensible to send the patient home, if they [i.e., the patients] will not make it [at home]. (Nurse, Department of medicine, FG 1)

In municipal social care, a common perception was that older adults were often prematurely discharged and that individual needs were not sufficiently considered. This resulted in these patients being quickly readmitted for the same reason. Overall, healthcare professionals in municipal social care had problems meeting the individual needs of older adults due to short preparation times:

D3: Yes, it is incredibly fast, since the new regulations came. It is a huge difference. OT3: Mm. They are discharged from the hospital and sent home the same day or the next day. If it’s not over a weekend, then. D3: Yes. It doesn’t feel like we are able to deliver a safe and secure homecoming for the patients that quickly. (Dialogue between occupational therapist and nurse/discharge coordinator, Municipality, FG 3)

Professionals in municipal social care, such as assistant nurses and occupational therapists, said that early assessments, the preparation of assistive devices, and quick follow-up visits, including at-home rehabilitation, were important in order to meet individual needs and prevent readmission. However, these interventions were considered difficult to plan or perform due to working time constraints and coordination difficulties in the older adult's home:

Rehabilitation and prevention take time … maybe it means that she [i.e., the older adult] should make her own porridge. That means, I need to make sure that she gets up out of bed in the morning, and then stands together with me by the stove. This time doesn’t exist … you don’t have those hours to be able to do it. Instead of being there for fifteen minutes, it might take half an hour, forty-five minutes to make breakfast with the older adult participating, and you don’t really have this opportunity. (Assistant nurse, Municipality, FG 3)

### A need for right competence among staff

An obstacle perceived in the various FGIs was a lack of personnel. The organization of healthcare and social care delivery for the older person via different healthcare providers was perceived as needing healthcare professionals with competence in geriatrics. Overall, the organization of home healthcare for the older person was considered stressful and poorly organized. Healthcare professionals in the homecare services described the work situation as strained and high pressure and not adapted to meeting care needs in older adults’ homes. They also had little or no contact with the physicians at the primary healthcare center:

The nurses in the homecare service do a great job, but they don’t have enough support from primary healthcare and they are very pressured. (Discharge coordinator/nurse, Department of medicine, FG 1)

Sometimes a patient was assessed as likely to benefit from a brief stay at a short-term care unit before going home. However, participants from primary healthcare and the municipality found that the care in these units was deficient in terms of a lack of registered nurses and physicians available round the clock:

We would benefit from expanding short-term care in the municipalities … It makes a big difference to have nurses [available] day and night, which would prevent readmission. (Physician, Primary healthcare, FG 2)

### Interprofessional coordination

This category describes the perception that coordination among healthcare providers concerning older adults and their relatives was important in care interventions. In particular, communication in connection with admission, discharge, and follow-up was considered important. Reasons for deficits in coordination and communication were assumed to partially stem from fragmented organization and a lack of interprofessional coordination within and among the healthcare providers.

### A need for interactive digital systems

The digital communication system between the different healthcare providers and the discharge coordinator at the hospital was described as a valuable tool that helped in maintaining contact. The information in this system was perceived as important for gaining a comprehensive view of the older adult's hospital stay and was also central to preparing care and the home environment for the older adult:

Communication is very important for this group of older adults. Communication among different physicians, among different healthcare professionals, but also a lot of communication with the patient. (Physician, Department of medicine, FG 2)

A shortcoming of this communication system was that not all healthcare professionals in municipal social care had the right to use the digital system. Also, the fact that an older adult had been readmitted was seldom communicated among healthcare providers during the DPP, which was said to be a deficiency:

They [i.e., acute care] have this information [about the readmission] in the medical records, but still, I sometimes find that they don’t act, even though they have the information about the readmission. (Nurse/discharge coordinator, Large municipality, FG 4)

### A need for closer integration

Physiotherapists said that they were highly dependent on the other healthcare professionals, such as occupational therapists, giving them information on the rehabilitation needs of older adults after discharge. On the other hand, most participants noted that initiatives including a specific homecare team (a small group of assistant nurses who meet older adults in their homes for the first 14 days after discharge, including contact with a physiotherapist, nurse, and occupational therapist) had emerged in some of the smaller municipalities and could be used to a greater extent. These homecare teams were described as contributing to continuity and safety at home and were assumed to prevent readmission.

The physicians at primary healthcare centers were perceived to contribute to preventing readmission by taking part in interprofessional teamwork and performing home visits on short notice after discharge, although this was not perceived to always be the case:

Physicians at primary healthcare centers could work in a more preventive and structured manner with medical rounds. Today, homecare nursing works differently at each primary healthcare center. For example, they could have regular rounds, not only have contact with a physician when medical needs arise. (Physician, Department of geriatrics, FG 2)

The care coordinator in primary healthcare was perceived as important in communicating preventive and upcoming individual care needs after a hospital stay. The care coordinator role should be established and used to a greater extent in primary healthcare:

There is a need for a ‘coordinator of frail older adults’ in primary healthcare, which wouldn’t be difficult to justify from an economic perspective. (Physician, Primary healthcare, FG 2)

According to the FGI participants, the structure and quality of the interprofessional work within and among healthcare providers varied. Basic interprofessional teamwork was seen as important but had deficits:

Nobody seems to be addressing this. Earlier, there were collaborative groups between the county council and the municipalities. They don’t exist anymore. There is no collaboration, it feels like it’s zero right now. We work in different areas, primary healthcare, we [i.e., emergency medical care], and the municipalities. (Discharge coordinator/nurse, Department of medicine, FG 1)

### Advanced care needs can be difficult to meet

This category, which includes perceptions that the older adults often had advanced care needs, is related to *resources* as well as *interprofessional coordination* and the further mentioned “how” aspects: *a need for time*, *a need for personnel with the right competence*, *a need for interactive digital systems*, and *a need for closer integration*. The advanced care needs could be described as difficult to meet and required, for example, time for investigation, competent staff, hospital beds, and collaboration.

Several older adults were described as having advanced care needs for further support from specialist care and contact with various healthcare professionals. Healthcare professionals in acute hospital care perceived that older adults’ reasons for readmission could vary, possibly including risk of falls, difficulties managing multiple medications, malnutrition, alcohol abuse, limited functional capacity, and a lack of social support. A febrile infection could also reduce the ability to cope at home. Multiple diagnoses, such as heart failure, chronic obstructive pulmonary disease, diabetes mellitus, mental illness, and cognitive impairment, were considered common factors contributing to readmission:

The idea was that we should identify these patients in the emergency room and then try to send them home instead of to a medical ward … and then you notice right away that it isn’t possible to send them home. It wasn’t that they were a little dizzy or needed a walker. Instead, they often have an illness that caused them to seek [emergency medical care]. (Healthcare coordinator/nurse, Hospital FG 1)

Healthcare professionals in both acute hospital care and the municipality perceived that readmission could occur regardless of the patient's age. In acute hospital care, nurses claimed that medical investigations and multiple care interventions from various healthcare professionals were important in order to meet individual care needs:

I usually say, but what is wrong with the patient? It’s not just that the older adults have suddenly realized [by themselves] that they have to be discharged. No, they have all these five basic diagnoses, and now heart failure and high blood sugar. (Nurse, Department of medicine, FG 1)

In hospital, various assessments and interventions need to be performed before discharge. These include medical investigations, adjustment of medications, mobilization, and sometimes trying out assistive devices. The use of structured assessments of ADL, fall risk, food intake, cognitive functions, pressure sores, etc., is considered to contribute to a suitable treatment plan and, it is hoped, will prevent readmission. Furthermore, older adults sometimes require adaptation of the home environment:

Performing various assessments before they are discharged is important, and many of us healthcare professionals are able to do this. … We have identified different needs during the hospital stay … and the occupational therapist and physiotherapist have performed an assessment of the patient’s ability related to the home situation. (Physician, Department of medicine, FG 2)

## Discussion

This study investigates healthcare professionals’ experiences of transitional care with respect to readmission in older adults. The results reveal that insufficient hospital beds, short hospital stays, insufficient staff, and the varied structure of interprofessional teamwork, respectively, were barriers to meeting advanced care needs and contributed to readmission. There is a need for the organizational structure during hospital stays and after discharge to be adapted to facilitate the care of older adults with advanced and multiple care needs.

In the *resources* category, the participants talked about the importance of available hospital beds, time, and personnel competence to be able to meet the advanced care needs of the older adults. The healthcare professionals said, for example, that hospital stays were often too short to satisfy the particular and often advanced care needs of the older adult. The unavailability of hospital beds was perceived as a barrier to meeting advanced care needs. Several studies have reported a negative relationship between length of hospital stay and the number of hospital beds, on one hand, and the risk of readmission, on the other (7, 43–45). In Sweden, the number of available hospital beds is 2.0 per 1000 inhabitants, which is the lowest per capita hospital bed rate in Europe (22). This is consistent with the results of this study, in which the participants said that short hospital stays increased the risk of unsafe care and early discharge from hospital. Moreover, low availability of hospital beds leads to difficulties in admitting patients from the emergency department (7). Furthermore, healthcare professionals in municipal social care regarded older adults as being discharged from hospital too quickly, which was seen as a barrier to meeting their advanced care needs. Short hospital stays lead to increased demands on primary healthcare and municipal social care. These demands are reinforced by the fact that places in nursing homes have been reduced by 30% since the year 2000 (38). Together with shortcomings in municipal social care, as mentioned in the previous paragraph, this leads to a vicious circle.

Another problem identified in relation to resources in the care chain was inadequate access to qualified medical staff in outpatient care. This may lead to individual medical care needs not being properly met (37). In addition, implementation of the “ageing in place” policy in Sweden and other European countries has led to stricter admission criteria for short-stay units and nursing homes (38). Advanced homecare is required to meet hospital standards, including qualified healthcare staff and access to laboratories on a 24 h basis (39). In reality, few units in Sweden can meet this standard.

According to the *interprofessional coordination* and *advanced care needs can be difficult to meet* categories, the FGI participants perceived that poor coordination among providers could lead to fragmented care, meaning that older adults receive incomplete assessment and treatment. One aspect mentioned in the FGIs was the need to enhance communication between healthcare providers and professionals. According to the participants, the lack of information from hospitals, and the fact that healthcare professionals were not permitted to use the communication system, led to difficulties in meeting individual and advanced care needs at home, increasing the risk of readmission to hospital. Previous studies have shown that communication often fails at hospital discharge (14, 35, 41). It is necessary to identify patients at risk of readmission, for example, by using a prediction score (47), to ensure that the right measures are taken at home (5). Such measures may ascertain the continuity of care interventions, ensure multidisciplinary teamwork, and stabilize the care chain (48, 49).

The healthcare professionals in the FGIs perceived that the multidisciplinary teamwork could be improved to better meet advanced care needs at home. The participants said, for example, that access to rehabilitation at home varied considerably. Close collaboration among healthcare providers is important in order to ensure that care interventions are implemented (8, 40, 41). To enable effective multidisciplinary collaboration, it is essential to have sufficient access to, for example, physicians, nurses, physiotherapists, and occupational therapists in both primary healthcare and municipal care (20, 42). This is a challenge in Sweden, where shortages of healthcare staff often lead to long waiting times and even lack of care (5). Similar issues may be observed in other countries as well, making it a challenge to ensure that healthcare and social care have the resources they need in order to provide qualified care.

One suggestion to improve coordination among healthcare providers was to establish an individualized discharge plan taking account of medical and functional needs, in order to address advanced care needs (8, 23, 34, 35). Such a plan would provide a basis for advanced care interventions in primary healthcare and municipal social care that is more relevant when caring for older adults with chronic conditions at home (36). The fragmentation of healthcare and municipal social care became more evident during the Covid-19 pandemic, showing the need for coordinated and efficient services from various healthcare providers (46). This is in line with our results, which indicate that individualized discharge plans should be used when older adults have had repeated short hospital stays, as their advanced care needs are probably not being met by routine methods.

### Methodological considerations

Due to the Covid-19 pandemic, only one FGI was held in person; the three other FGIs were held remotely, which may have influenced the interaction and the sharing of individual perceptions and ideas. However, the risks of a sense of relative anonymity, of increased absenteeism, and of the rescheduling of remote meetings, as mentioned in a previous study (50), did not differ between the FGIs. According to Janghorban et al. (50), information from in-person FGIs is comparable to that from digital or telephone interviews; this was also observed in this study, in which data from all groups displayed similar levels of richness and variation.

As researchers may influence both data collection and analysis, the understandings and interpretations of the data were regularly discussed among the authors during the research process. The prior experience of the first author, who has encountered older adults and healthcare professionals in the acute medical care field, might have facilitated an understanding of the phenomenon; however, this experience could also have threatened the confirmability and trustworthiness. To reduce the risk of bias and strengthen trustworthiness, the first and last authors’ analyses involved all authors, in a form of triangulation. In addition, quotations have been used to illustrate the empirical data and the descriptive categories, which strengthens trustworthiness and confirmability (51). Our analysis also indicated that similar experiences recurred across the various FGIs. Nonetheless, it is possible that collecting further data by conducting additional FGIs or altering the participant composition might have yielded supplementary insights or nuanced perceptions not captured in the FGIs conducted. The transferability of this study may be limited, as healthcare systems differ between countries.

## Conclusion

There are several interacting reasons why readmissions of older adults are common, the most important being premature hospital discharge due to lack of beds and staff. As well, onward reporting between hospital and outpatient healthcare providers is poor and there is a lack of qualified staff in the home setting. To prevent readmission, medical care and multidisciplinary teamwork must be improved in the home setting. Only in this way can aging in place be implemented when it comes to seriously ill older adults.

## Data Availability

The original contributions presented in the study are included in the article/Supplementary Material, further inquiries can be directed to the corresponding author.

## References

[1] Baxter R O’Hara J Murray J Sheard L Cracknell A Foy R Partners at care transitions: exploring healthcare professionals’ perspectives of excellence at care transitions for older people. BMJ open. (2018) 8(9):e022468. 10.1136/bmjopen-2018-022468 30232111 PMC6150145

[2] Bångsbo A Dunér A Lidén E. Patient participation in discharge planning conference. Int J Integr Care. (2014) 14:e030. 10.5334/ijic.1543 25411572 PMC4236306

[3] Nordmark S Söderberg S Skär L. Information exchange between registered nurses and district nurses during the discharge planning process: cross-sectional analysis of survey data. Inform Health Soc Care. (2015) 40(1):23–44. 10.3109/17538157.2013.872110 24393036

[4] Deschodt M Devriendt E Sabbe M Knockaert D Deboutte P Boonen S Characteristics of older adults admitted to the emergency department (ED) and their risk factors for ED readmission based on comprehensive geriatric assessment: a prospective cohort study. BMC Geriatr. (2015) 15:54. 10.1186/s12877-015-0055-7 25928799 PMC4417280

[5] Socialstyrelsen [The National Board of Health and Welfare]. Återinskrivningar av Multisjuka och Sköra äldre [Readmissions of Multi-diseased and Frail Older Adults]. Stockholm: The National Board of Welfare (2021). Available online at: https://www.socialstyrelsen.se/om-socialstyrelsen/pressrum/press/var-fjarde-sjukhusvistelse-bland-de-mest-sjuka-aldre-foljs-av-en-ny-inskrivning-inom-kort/ (Accessed November 5, 2021).

[6] Berry JG Gay JC Joynt Maddox K Coleman EA Bucholz EM O’Neill MR Age trends in 30 day hospital readmissions: US national retrospective analysis. Br Med J. (2018) 360:k497. 10.1136/bmj.k497 29487063 PMC5827573

[7] Fabbian F Boccafogli A De Giorgi A Pala M Salmi R Melandri R The crucial factor of hospital readmissions: a retrospective cohort study of patients evaluated in the emergency department and admitted to the department of medicine of a general hospital in Italy. Eur J Med Res. (2015) 20:6. 10.1186/s40001-014-0081-5 25623952 PMC4314760

[8] Goncalves-Bradley DC Lannin NA Clemson L Cameron ID Shepperd S. Discharge planning from hospital. Cochrane Database Syst Rev. (2022) 2(2):CD000313. 10.1002/14651858.CD000313.pub5 35199849 PMC8867723

[9] Howard-Anderson J Busuttil A Lonowski S Vangala S Afsar-Manesh N. From discharge to readmission: understanding the process from the patient perspective. J Hosp Med. (2016) 11(6):407–12. 10.1002/jhm.2560 26895238

[10] Park LA D Mastey A Sun J Hicks J. Institution specific risk factors for 30 day readmission at a community hospital: a retrospective observational study. BMC Health Serv Res. (2014) 14(40):1–6. 10.1186/1472-6963-14-40 24467793 PMC3916302

[11] Alders P Schut FT. Trends in ageing and ageing-in-place and the future market for institutional care: scenarios and policy implications. Health Econ Policy Law. (2019) 14(1):82–100. 10.1017/S1744133118000129 29779497

[12] Grabowski DC. The future of long-term care requires investment in both facility- and home-based services. Nat Aging. (2021) 1(1):10–1. 10.1038/s43587-020-00018-y 37117999

[13] Storm M Siemsen IM Laugaland K Dyrstad DN Aase K. Quality in transitional care of the elderly: key challenges and relevant improvement measures. Int J Integr Care. (2014) 14:e013. 10.5334/ijic.1194 24868196 PMC4027895

[14] Di Pollina L Guessous I Petoud V Combescure C Buchs B Schaller P Integrated care at home reduces unnecessary hospitalizations of community-dwelling frail older adults: a prospective controlled trial. BMC Geriatr. (2017) 17(1):53. 10.1186/s12877-017-0449-9 28196486 PMC5310012

[15] Laugaland K Aase K Barach P. Interventions to improve patient safety in transitional care–a review of the evidence. Work (Reading, Mass). (2012) 41(Suppl 1):2915–24. 10.3233/wor-2012-0544-2915 22317162

[16] Shepperd S Lannin NA Clemson LM McCluskey A Cameron ID Barras SL. Discharge planning from hospital to home. Cochrane Database Syst Rev. (2013) 1:Cd000313. 10.1002/14651858.CD000313.pub4 23440778

[17] Benzo R Vickers K Novotny PJ Tucker S Hoult J Neuenfeldt P Health coaching and chronic obstructive pulmonary disease rehospitalization. A randomized study. Am J Respir Crit Care Med. (2016) 194(6):672–80. 10.1164/rccm.201512-2503OC 26953637 PMC5027231

[18] Kociol RD Peterson ED Hammill BG Flynn KE Heidenreich PA Pina IL National survey of hospital strategies to reduce heart failure readmissions: findings from the get with the guidelines-heart failure registry. Circ Heart Fail. (2012) 5(6):680–7. 10.1161/CIRCHEARTFAILURE.112.967406 22933525

[19] Kripalani S Theobald CN Anctil B Vasilevskis EE. Reducing hospital readmission rates: current strategies and future directions. Annu Rev Med. (2014) 65:471–85. 10.1146/annurev-med-022613-090415 24160939 PMC4104507

[20] Leppin AL Gionfriddo MR Kessler M Brito JP Mair FS Gallacher K Preventing 30-day hospital readmissions: a systematic review and meta-analysis of randomized trials. JAMA Intern Med. (2014) 174(7):1095–107. 10.1001/jamainternmed.2014.1608 24820131 PMC4249925

[21] Rogers AT Bai G Lavin RA Anderson GF. Higher hospital spending on occupational therapy is associated with lower readmission rates. Med Care Res Rev. (2016) 6:668–86. 10.1177/1077558716666981 27589987

[22] Organization for Economic Co-operation and Development [OECD]. Health at a Glance: Europe State of Health in the EU Cycle. Stockholm: OECD publishing 2020 (2023). Available online at: https://www.oecd.org/health/health-at-a-glance-europe/ (Accessed March 24, 2023)

[23] Ministry of Social Affairs [Swedish Parliament]. Socialdepartementet [Ministry of Social Affairs] Lag (2017:612) om Samverkan vid Utskrivning Från Sluten Hälso- och Sjukvård [Law (2017:612) on Collaborations at Discharge from Hospital]. Stockholm: Ministry of Social Affairs (2017). Available online at: https://www.riksdagen.se/sv/dokument-lagar/dokument/svensk-forfattningssamling/lag-2017612-om-samverkan-vid-utskrivning-fran_sfs-2017-612

[24] Dilworth S Higgins I Parker V. Feeling let down: an exploratory study of the experiences of older people who were readmitted to hospital follwing discharge. Contemp Nurse. (2012) 42(2):280–8. 10.5172/conu.2012.42.2.280 23181378

[25] Greysen SR Harrison JD Kripalani S Vasilevskis E Robinson E Metlay J Understanding patient-centred readmission factors: a multi-site, mixed-methods study. BMJ Qual Saf. (2017) 26(1):33–41. 10.1136/bmjqs-2015-004570 26769841 PMC11907771

[26] Jonsson M Fredriksson C Holmefur M. Everyday activities at home—experiences of older repeatedly readmitted people. Scand J Occup Ther. (2020) 29(7):555–62. 10.1080/11038128.2020.1849393 33222567

[27] Marton F. Phenomenography—describing conceptions of the world around US. Instr Sci. (1981) 10:177–200. 10.1007/BF00132516

[28] Kreuger R Casey M. Focus Groups—a Practical Guide for Applied Research. 5th Ed Thousand Oaks, California: SAGE publications (2015).

[29] Ouchi Y Rakugi H Arai H Akishita M Ito H Toba K Redefining the elderly as aged 75 years and older: proposal from the joint committee of Japan gerontological society and the Japan geriatrics society. Geriatr Gerontol Int. (2017) 17(7):1045–7. 10.1111/ggi.13118 28670849

[30] Stenfors-Hayes T Hult H Dahlgren MA. A phenomenographic approach to research in medical education. Med Educ. (2013) 47(3):261–70. 10.1111/medu.12101 23398012

[31] Dahlgren L Fallsberg M. Phenomenography as a qualitative approach in social pharmacy research. J Soc Adm Pharm. (1991) 8:150–6.

[32] Edlundh BM McDougall AG. Allt om NVivo 11. Stockholm: Form & Kunskap (2017). p. 372.

[33] Hadi MA Jose Closs S. Ensuring rigour and trustworthiness of qualitative research in clinical pharmacy. Int J Clin Pharm. (2016) 38(3):641–6. 10.1007/s11096-015-0237-6 26666909

[34] Courtney M Edwards H Chang A Parker A Finlayson K Hamilton K. Fewer emergency readmissions and better quality of life for older adults at risk of hospital readmission: a randomized controlled trial to determine the effectiveness of a 24-week exercise and telephone follow-up program. J Am Geriatr Soc. (2009) 57(3):395–402. 10.1111/j.1532-5415.2009.02138.x 19245413

[35] Leland NE Roberts P De Souza R Sun Hwa C Shah K Robinson M. Care transition processes to achieve a succesful community discharge after postacute care: a scoping review. Am J Occup Ther. (2019) 73:1–9. 10.5014/ajot.2019.005157 30839269

[36] Gruneir A Silver MJ Rochon PA. Emergency department use by older adults: a literature review on trends, appropriateness, and consequences of unmet health care needs. Med Care Res Rev. (2011) 68(2):131–55. 10.1177/1077558710379422 20829235

[37] Ernsth Bravell M Bennich M Walfridsson C. “In August, I counted 24 different names”: swedish older Adults’ experiences of home care. J Appl Gerontol. (2021) 40(9):1020–8. 10.1177/0733464820917568 32418462 PMC8299777

[38] Johansson L Schön P. Quality and Cost-effictiveness in Longterm Care and Dependency Prevention. Stockholm: CEQUA LTC network Country report Sweden (2017). Available online at: https://aldrecentrum.se/wp-content/uploads/2020/07/quality_and_cost-effectiveness_in_long-term_care_and_d.pdf (Accessed November 8, 2020)

[39] Socialstyrelsen [The National Board of Health and Welfare]. God och Nära Vård [Safe and Close Care]. Stockholm: The National Board of Welfare (2019). Available online at: https://www.socialstyrelsen.se/kunskapsstod-och-regler/omraden/god-och-nara-vard/ (Accessed November 5, 2021)

[40] Frechman E Dietrich MS Walden RL Maxwell CA. Exploring the uptake of advance care planning in older adults: an integrative review. J Pain Symptom Manage. (2020) 60(6):1208–22.e59. 10.1016/j.jpainsymman.2020.06.043 32645455 PMC7342022

[41] Hesselink G Schoonhoven L Plas M Wollersheim H Vernooij-Dassen M. Quality and safety of hospital discharge: a study on experiences and perceptions of patients, relatives and care providers. Int J Qual Health Care. (2013) 25(1):66–74. 10.1093/intqhc/mzs066 23184652

[42] Socialdepartementet [Ministry of Health and Social Affairs]. Läs mig! Nationell Kvalitetsplan för Vård och Omsorg för äldre Personer [Read me! National Plan for Health Care and Social Services for Older People] Stockholm: The National Board of Welfare (2017). Available online at: http://www.regeringen.se/rattsdokument/statens-offentliga-utredningar/2017/03/sou-201721/ (Accessed October 3, 2018)

[43] Ashton CM Wray NP. A conceptual framework for the study of early readmission as an indicator of quality of care. Soc Sci Med. (1996) 43(11):1533–41. 10.1016/S0277-9536(96)00049-4 8961397

[44] Magdelijns FJ Schepers L Pijpers E Stehouwer CD Stassen PM. Unplanned readmissions in younger and older adult patients: the role of healthcare-related adverse events. Eur J Med Res. (2016) 21(1):35. 10.1186/s40001-016-0230-0 27634174 PMC5025596

[45] Moran J Colbert CY Song J Hull J Rajan S Varghees S Residents examine factors associated with 30-day, same-cause hospital readmissions on an internal medicine service. Am J Med Qual. (2013) 28(6):492–501. 10.1177/1062860613480827 23550215

[46] Socialdepartementet[Ministry of Social Affairs]. The Care in Older Adults in Pandemic 2020. Stockholm: Ministry of Social Affairs (2020). Available online at: https://www.regeringen.se/rattsliga-dokument/statens-offentliga-utredningar/2020/12/sou-202080/ (Accessed March 24, 2023)

[47] Robinson R. The HOSPITAL score as a predictor of 30 day readmission in a retrospective study at a university affiliated community hospital. PeerJ. (2016) 4:e2441. 10.7717/peerj.2441 27651999 PMC5018668

[48] Arsenault-Lapierre G Henein M Gaid D Le Berre M Gore G Vedel I. Hospital-at-home interventions vs in-hospital stay for patients with chronic disease who present to the emergency department: a systematic review and meta-analysis. JAMA Netw Open. (2021) 4(6):e2111568. 10.1001/jamanetworkopen.2021.11568 34100939 PMC8188269

[49] Facchinetti G D’Angelo D Piredda M Petitti T Matarese M Oliveti A Continuity of care interventions for preventing hospital readmission of older people with chronic diseases: a meta-analysis. Int J Nurs Stud. (2020) 101:103396. 10.1016/j.ijnurstu.2019.103396 31698168

[50] Janghorban R Latifnejad Roudsari R Taghipour A. Skype interviewing: the new generation of online synchronous interview in qualitative research. Int J Qual Stud Health Well Being. (2014) 9:24152. 10.3402/qhw.v9.24152 24746247 PMC3991833

[51] Lincoln YS Guba EG. Naturalistic Inquiry. Beverly Hills, CA: Sage (1985).

